# Evaluation and Treatment of Vascular Cognitive Impairment by Transcranial Magnetic Stimulation

**DOI:** 10.1155/2020/8820881

**Published:** 2020-10-27

**Authors:** Mariagiovanna Cantone, Giuseppe Lanza, Francesco Fisicaro, Manuela Pennisi, Rita Bella, Vincenzo Di Lazzaro, Giovanni Di Pino

**Affiliations:** ^1^Department of Neurology, Sant'Elia Hospital, ASP Caltanissetta, Caltanissetta 93100, Italy; ^2^Department of Surgery and Medical-Surgical Specialties, University of Catania, Catania 95123, Italy; ^3^Department of Neurology IC, Oasi Research Institute–IRCCS, Troina 94108, Italy; ^4^Department of Biomedical and Biotechnological Sciences, University of Catania, Catania 95123, Italy; ^5^Department of Medical and Surgical Sciences and Advanced Technologies, University of Catania, Catania 95123, Italy; ^6^Unit of Neurology, Neurophysiology, Neurobiology, Department of Medicine, Università Campus Bio-Medico di Roma, Rome 00128, Italy; ^7^Research Unit of Neurophysiology and Neuroengineering of Human-Technology Interaction (NeXTlab), Università Campus Bio-Medico di Roma, Rome 00128, Italy

## Abstract

The exact relationship between cognitive functioning, cortical excitability, and synaptic plasticity in dementia is not completely understood. Vascular cognitive impairment (VCI) is deemed to be the most common cognitive disorder in the elderly since it encompasses any degree of vascular-based cognitive decline. In different cognitive disorders, including VCI, transcranial magnetic stimulation (TMS) can be exploited as a noninvasive tool able to evaluate *in vivo* the cortical excitability, the propension to undergo neural plastic phenomena, and the underlying transmission pathways. Overall, TMS in VCI revealed enhanced cortical excitability and synaptic plasticity that seem to correlate with the disease process and progression. In some patients, such plasticity may be considered as an adaptive response to disease progression, thus allowing the preservation of motor programming and execution. Recent findings also point out the possibility to employ TMS to predict cognitive deterioration in the so-called “brains at risk” for dementia, which may be those patients who benefit more of disease-modifying drugs and rehabilitative or neuromodulatory approaches, such as those based on repetitive TMS (rTMS). Finally, TMS can be exploited to select the responders to specific drugs in the attempt to maximize the response and to restore maladaptive plasticity. While no single TMS index owns enough specificity, a panel of TMS-derived measures can support VCI diagnosis and identify early markers of progression into dementia. This work reviews all TMS and rTMS studies on VCI. The aim is to evaluate how cortical excitability, plasticity, and connectivity interact in the pathophysiology of the impairment and to provide a translational perspective towards novel treatments of these patients. Current pitfalls and limitations of both studies and techniques are also discussed, together with possible solutions and future research agenda.

## 1. Introduction

### 1.1. Vascular Cognitive Impairment

Globally, vascular cognitive impairment (VCI) is defined as a decline in cognition due to cerebrovascular injury. It is currently viewed as an “umbrella term” encompassing mild VCI, vascular dementia (VaD), and mixed dementia [1–3]. Mild VCI is a decline in cognition not fully satisfying the diagnostic criteria for dementia [4, 5]. VaD identifies cognitively impaired patients who have lost their functional independence due to vascular lesions and includes different subtypes, such as poststroke dementia, multi-infarct dementia, strategic infarct dementia, and the subcortical ischemic VaD. Finally, mixed dementia is the result of both vascular and degenerative pathophysiology, most commonly of Alzheimer's disease- (AD-) type [6]. Hence, VCI is deemed to be the most common cognitive disorder in the elderly, with a growing impact on patients' quality of life (QoL) and on social and healthcare system [2]. Moreover, vascular-derived impairment has a great prevalence in all types of cognitive decline, where its contribution to the deficits is considerable. Of note, this is the only contribution that can be, at least in part, treatable and preventable [7, 8].

In addition to the affected cognitive domains, which typically are attention, processing speed, and executive functioning [9], VCI can impact also on several neuropsychiatric aspects, such as behavioral and mood disturbances, making this disorder extremely heterogeneous [10–12]. Apathy, irritability, disinhibition, and psychomotor retardation are common examples of the behavioral changes found in VCI patients, while depression is the most reported mood disorder. Behavioral and mood changes correlate with the worsening of cognitive and functional status and significantly reduce the QoL of patients and caregivers [13–15].

The pathophysiology accounting for cognitive and behavioral-mood dysfunction in VCI is still not completely defined. The so-called “disconnection hypothesis,” based on the analysis of brain images of large samples [16, 17], points to the result of a “disruption” of cortical and/or subcortical loops implicated in cognition and mood-affect regulation, due to acute of chronic cerebrovascular lesions [18–20]. In magnetic resonance imaging (MRI) of stroke or cerebral small vessel disease, ischemic white matter lesions (WMLs) are clinically associated with cognitive impairment [21]. In large community-based populations [16, 22, 23], WMLs are also associated with nonmotor sequelae, and cognitive and mood-behavior impairment was especially linked with the ischemic disruption of the prefrontal cortical-subcortical circuits [24]. In stroke survivors, the atrophy of the medial temporal lobe predicts early cognitive dysfunction [25]. Even subcortical ischemic vascular disease, including silent lacunar infarcts and WMLs, is associated with executive dysfunction and late-life depression, which is a clinical and neuroimaging condition now referred as “vascular depression” [20]. Taken together, it has been clearly established that cognitive limitations and depressive disorders are tightly intertwined in patients with both acute and chronic cerebrovascular diseases, such as stroke and small vessel disease, respectively [9, 11, 13, 26].

VCI diagnosis must capitalize from clinical and neuropsychological evaluations, as well as from structural and functional neuroimaging [11]. However, the search for novel hallmarks of disease process and progression, such as serological, cerebrospinal fluid (CSF), and instrumental markers, is needed to allow an early, tailored, and accurate screening of VCI patients. This will also pave the way to innovative therapeutic strategies and to the identification of predictors of drug response [27, 28]. Moreover, the noninvasively investigation of cortical circuitry in VCI patients has produced intriguing findings on abnormal cortical connectivity [29] and plasticity [30, 31].

Overall, neural plasticity refers to the brain's ability, particularly of the cerebral cortex, of reorganizing and adapting to different constantly changing environmental stimuli. This takes place through phenomena of modification of synaptic connection strength (like long-term potentiation (LTP) and long-term depression (LTD)), modification of the representation pattern and neuronal activity, modulation of gene induction and expression, changes in cerebral blood flow, and neurotrophin release [32]. Neural plasticity is an essential substrate for learning and memory [33], and its involvement in dementia (such as AD), movement disorders (such as Parkinson's disease), and neuropsychiatric disorders (such as major depression) [34] is well documented. Although abnormalities in brain plasticity and its components have been widely demonstrated in dementia, their role in the pathophysiology of VCI and in the counteraction against disease progression is still not understood. In this scenario, the contribution of noninvasive and translational brain stimulation techniques, namely, transcranial magnetic stimulation (TMS), is becoming of pivotal importance.

### 1.2. Transcranial Magnetic Stimulation

From the pioneering application of TMS to assess the primary motor cortex (M1) and the cortical-spinal conductivity [35], scientists boost the potentialities of this technique, which is employed today to study cortical excitability, to map connectivity, and to probe the propensity to undergo plastic phenomena [36]. This gives novel insights into the pathophysiology underlying several neurological and neuropsychiatric diseases [37, 38].

A functional assessment of global cortical excitability and cortico-spinal conduction results from the application of single magnetic pulses at adequate stimulator intensity over the M1 that elicits a motor evoked potential (MEP) recordable on the contralateral target muscle [39, 40]. The MEP amplitude is an aggregate measure of the excitation state of M1's output cells, nerve roots, and the conduction along the peripheral motor nerves till the muscles [41]. The resting motor threshold (rMT), i.e., the minimum intensity of stimulation needed to evoke a MEP, is a basic index of M1 excitability, as it is a compound measure of the membrane excitability of cortical neurons, the neural inputs into pyramidal cells within the cortex, and the excitability of spinal motor neurons, neuromuscular junctions, and muscles [42].

During a tonic contraction of the contralateral muscles, the result of a suprathreshold TMS pulse applied to the M1 is a few hundred milliseconds suppression of the electromyographic (EMG) activity of those muscles [43]. This phenomenon, called contralateral cortical silent period (cSP), is exploited as a functional measure of intracortical inhibitory circuits [44, 45], mainly mediated by the gamma-aminobutyric acid- (GABA-) B transmission [46]. Conversely, activation of the muscle and stimulation of the hemisphere of the same side evoke the ipsilateral silent period (iSP), which it is thought to receive some modulated effects from transcallosal output neurons that project to contralateral GABAergic interneurons [47, 48].

Paired-pulse TMS paradigm allows the assessment of the short-interval intracortical inhibition (SICI) and the intracortical facilitation (ICF) of the motor response [49, 50]. The activity of GABA-A interneurons is the most likely mediator of SICI [51, 52], whereas the neurophysiology of ICF is more complex. It probably relates to the activation of a cortical circuit projecting upon cortico-spinal cells different from that preferentially activated by single-pulse TMS. ICF seems dependent to a great extent on the activity of glutamatergic excitatory interneurons, although other mediators are known to contribute [53, 54].

Researchers have also the possibility of investigating the sensory-motor interactions in the cerebral cortex by using specific TMS protocols. One of these allows for the investigation of the short-latency afferent inhibition (SAI), which mainly reflects the central cholinergic circuits' integrity [55]. Indeed, while the muscarinic antagonistic scopolamine in healthy subjects reduces or abolishes SAI [56], acetylcholine positively modulates it [57]. It has been suggested that SAI may depend on the integrity of circuits linking sensory input and motor output [58, 59], thus providing valuable diagnostic information in a variety of cognitive and movement disorders [60, 61]. Finally, TMS also allows the study of synaptic plasticity through different paradigms of paired-associative stimulation (PAS), e.g., by applying a magnetic stimulus after a brief period of exercise or by using repetitive low-frequency median nerve stimulation combined with TMS over the contralateral M1 [62]. PAS has shown to lead to LTP-/LTD-like changes within the sensory-motor pathways [63].

**Figure 1:**
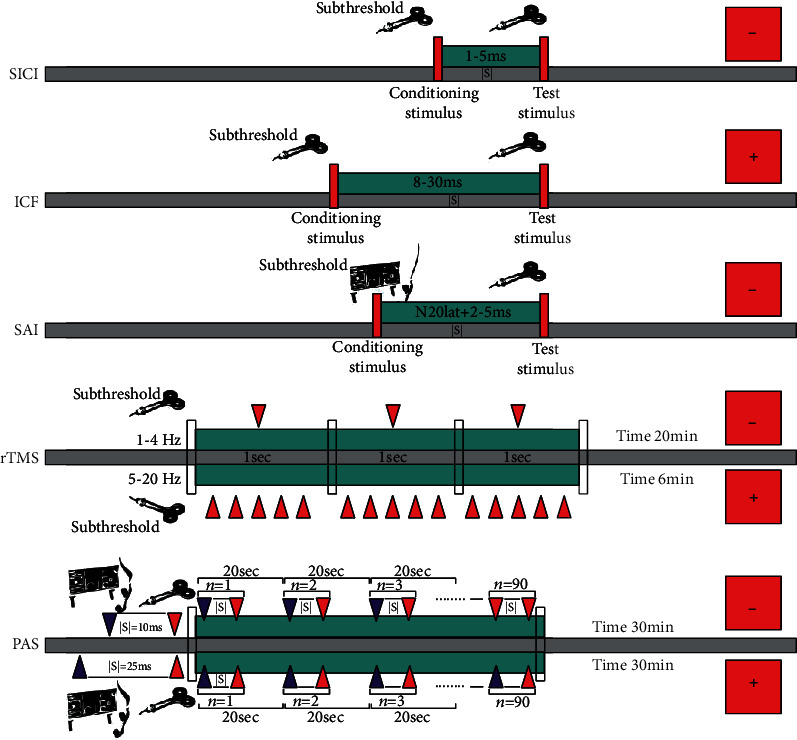
Schematic representation of some TMS measures and protocols of stimulation. Legend (in alphabetic order): ICF: intracortical facilitation; ISI: interstimulus interval; SAI: short-latency afferent inhibition; PAS: paired-associative stimulation; rTMS: repetitive transcranial magnetic stimulation; SICI: short-interval intracortical inhibition; +: facilitatory/excitatory effect; -: inhibitory/suppressive effect.

Figure 1 schematically illustrates the technical aspects and the neurophysiological correlate of SICI, ICF, SAI, PAS, and repetitive TMS (rTMS).

### 1.3. Repetitive TMS

Repetitive TMS (rTMS) over the same cortical target induces a transient modification of the cortex excitability, which decreases by using low frequencies (≤1 Hz) and increases by using high frequencies (5-20 Hz) [64]. The neurobiology of rTMS seems to share many features with LTD and LTP's induction by tetanic stimulation in cortical slices [65], such as the dependence from N-methyl-D-aspartate- (NMDA-) receptor activity [66], the sensibility to prior synaptic activation [67], and the strict link with stimulation frequency [68]. The short-term changes in synaptic efficacy and the rapid downregulation of GABA-related inhibitory circuits are key processes of calcium- and sodium channel-dependent LTP plasticity [69, 70]. Conversely, by inducing LTD-like responses, rTMS decreases the synaptic efficacy [71, 72].

The effects of repeated sessions of rTMS persist in time and act by enhancing plasticity when needed but also by downregulating it when plasticity becomes inappropriate or even maladaptive [73]. For all those reasons, the translational therapeutic and rehabilitative applications of rTMS may cover a wide range of neurological and psychiatric disorders [74, 75]. Accordingly, in October 2008, the Food and Drug Administration (FDA) approved rTMS as an add-on treatment of adult drug-resistant major depressive disorder (MDD). Besides, specific rTMS paradigms, like the theta-burst stimulation [76] or the quadripulse stimulation [77], may help in a better comprehension of synaptic plasticity phenomena or even more complex responses, such as the metaplasticity (i.e., “plasticity of synaptic plasticity”) [78–80].

Overall, rTMS is safe and well tolerated. A discomfort caused by scalp or facial muscle twitching and transient headache are the most commonly reported side effects [81], while the induction of seizures is a very rare but serious adverse effect, although not common even employing supratherapeutic stimulations [82]. Nevertheless, epileptic patients or those with risk factors of epilepsy should be managed with extreme caution.

### 1.4. Aim e Rationale

To date, the exact relationship between cognitive functioning, motor cortical excitability, and synaptic plasticity in VCI is not completely unveiled. In this work, we review all the TMS and rTMS studies related to VCI to provide a timely translational perspective on how cortical excitability and network connectivity interact to determine the pathophysiology and plastic changes in VCI and its subtypes, and how these findings may be exploited by experimental treatments. Current pitfalls and limitations of both studies and techniques are also discussed, together with possible solutions and future research agenda.

## 2. Methods

A literature search was carried out to find all the relevant studies of TMS and rTMS in VCI. A PubMed-based literature review was performed by using the following search queries:

- (“transcranial magnetic stimulation” [MeSH Terms] OR (“transcranial” [All Fields] AND “magnetic” [All Fields] AND “stimulation” [All Fields]) OR “transcranial magnetic stimulation” [All Fields] OR (“repetitive” [All Fields] AND “transcranial” [All Fields] AND “magnetic” [All Fields] AND “stimulation” [All Fields]) OR “repetitive transcranial magnetic stimulation” [All Fields]) AND “vascular” [All Fields] AND (“cognitive dysfunction” [MeSH Terms] OR (“cognitive” [All Fields] AND “dysfunction” [All Fields]) OR “cognitive dysfunction” [All Fields] OR (“cognitive” [All Fields] AND “impairment” [All Fields]) OR “cognitive impairment” [All Fields])
- (“transcranial magnetic stimulation” [MeSH Terms] OR (“transcranial” [All Fields] AND “magnetic” [All Fields] AND “stimulation” [All Fields]) OR “transcranial magnetic stimulation” [All Fields] OR (“repetitive” [All Fields] AND “transcranial” [All Fields] AND “magnetic” [All Fields] AND “stimulation” [All Fields]) OR “repetitive transcranial magnetic stimulation” [All Fields]) AND (“dementia, vascular” [MeSH Terms] OR (“dementia” [All Fields] AND “vascular” [All Fields]) OR “vascular dementia” [All Fields])
- (“transcranial magnetic stimulation” [MeSH Terms] OR (“transcranial” [All Fields] AND “magnetic” [All Fields] AND “stimulation” [All Fields]) OR “transcranial magnetic stimulation” [All Fields] OR (“repetitive” [All Fields] AND “transcranial” [All Fields] AND “magnetic” [All Fields] AND “stimulation” [All Fields]) OR “repetitive transcranial magnetic stimulation” [All Fields]) AND “vascular” [All Fields] AND (“depressive disorder” [MeSH Terms] OR (“depressive” [All Fields] AND “disorder” [All Fields]) OR “depressive disorder” [All Fields] OR “depression” [All Fields] OR “depression” [MeSH Terms])
- (“transcranial magnetic stimulation” [MeSH Terms] OR (“transcranial” [All Fields] AND “magnetic” [All Fields] AND “stimulation” [All Fields]) OR “transcranial magnetic stimulation” [All Fields] OR (“repetitive” [All Fields] AND “transcranial” [All Fields] AND “magnetic” [All Fields] AND “stimulation” [All Fields]) OR “repetitive transcranial magnetic stimulation” [All Fields]) AND (“cadasil” [MeSH Terms] OR “cadasil” [All Fields])

Two independent authors (FF and MP) screened titles and abstracts of all retrieved publications, and disagreements were solved by the consensus of a third author (RB). Duplicated entries, retracted publications, studies on other diseases different from VCI or its subtypes, works on animals or *in vitro*, studies without statistical analysis, non-English written papers, publications that are not research studies (i.e., commentaries, letters, editorials, and reviews), and any other article that did not fit with the scope of this review were excluded. Articles listed in the references were also reviewed in search of more data. We considered studies indexed from the database inception to April 2020.

## 3. Results and Discussion

**Figure 2:**
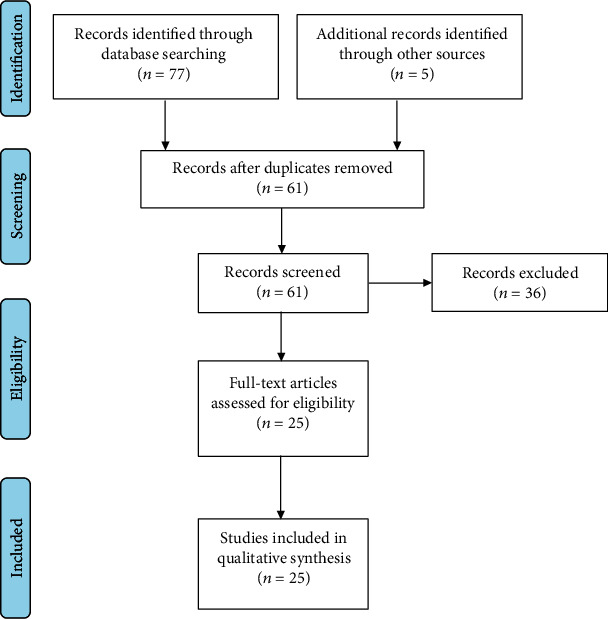
Flow diagram showing the search strategy, the number of records identified, and the number of included/excluded studies [106]. This figure is reproduced from Moher, David et al. preferred reporting items for systematic reviews and meta-analyses: the PRISMA statement. BMJ 2009; 339:b2535 (under the Creative Commons Attribution License/public domain).

A total of 77 results were originally retrieved. Of these, 20 peer-reviewed publications were selected according to the above inclusion and exclusion criteria. The examination of the references from relevant papers detected 5 additional studies fitting the purpose of this review. Eventually, a total of 25 papers were included (Figure 2), and their main findings were analyzed clustering within two groups, one on TMS studies (summarized in Table 1) and the other on rTMS studies (summarized in Table 2). More in details, we included in the TMS group 6 studies on mild VCI [30, 31, 83–86], 6 on VaD [87–92], 3 on vascular depression [93–95], and 4 on cerebral autosomal dominant arteriopathy with subcortical infarcts and leukoencephalopathy (CADASIL) [96–99], while the rTMS studies group consisted of 2 articles in mild VCI [100, 101] and 4 in vascular depression [102–105].

### 3.1. Mild Vascular Cognitive Impairment

The identification of mild VCI subjects at risk for clinical progression into VaD or mixed dementia is a crucial challenge for both clinicians and researchers because it may raise the chances to early diagnose and to delay the disease progression.

A previous study on nondemented elderly patients with subcortical ischemic vascular disease and clinical-cognitive profile of mild VCI [30] found that prefrontal subcortical loops lesioned by the ischemic interruption due to WMLs or lacunar infarcts lead to functional changes of the intracortical excitatory neuronal circuits (i.e., increased ICF). In this patient class, a further study has also shown that iSP is spared [83], unlike neurodegenerative disorders, such as AD and mild cognitive impairment (MCI), that show abnormal iSP since the early stages. This suggests a functional integrity of the transcallosal inhibitory connections in VCI, at least in the early phase [107].

A TMS study carried out on the same participants after a 2-year follow-up [31] found that, compared to the baseline, patients exhibited an increased global cortical excitability (reduction of the median rMT) and a significant worsening of the score of neuropsychological tests evaluating the frontal lobe abilities. The researchers interpreted the findings as indicative of plastic compensatory mechanisms in response to cortical disconnection [31, 108]. In particular, the study hypothesizes that rMT might become abnormal when VCI progresses to VaD and that its value can be used as a “neurophysiological cut-off” segregating patients who will progress from those who will remain cognitively stable. It is known, indeed, that higher motor cortex facilitation marks higher risk to convert from normal aging brain to cognitive impairment up to an overt dementia [31]. These findings are in line with the observation of enhanced cortical plasticity and reorganization, probably as compensatory mechanisms due to impaired cerebral autoregulation, in nondemented patients with severe ischemic small vessel disease [84, 85].

Notably, mild VCI individuals do not show impaired cholinergic activity compared to age-matched controls [86], which might suggest a distinctive cholinergic profile characterizing the early stages of VaD and differentiating it from the “cholinergic” forms of cognitive decline, such as MCI and AD [109]. However, cholinergic involvement in VaD is still under debate, and the few available TMS data show conflicting results [88, 89, 91]. The high heterogeneity in the location and severity of subcortical infarcts, leading to variations in the resultant distribution and magnitude of the cholinergic denervation, may be a reasonable explanation [91]. Finally, SAI might be useful in the identification of responders to the acetylcholinesterase inhibitors and, indirectly, in the differentiation between “cholinergic” and “non-cholinergic” forms of dementia [86].

### 3.2. Vascular Dementia

A common feature of AD and VaD patients is the increase of M1 excitability, (i.e., reduction of rMT), which differentiate them from normal brain aging [87]. Different studies converge on the hypothesis that an enhanced excitability and plasticity seems to have a role in counteracting cognitive decline in the elderly [110] as a compensatory response to neuronal loss and vascular injury [111]. However, this likely hypothesis warrants future experimental investigations on longitudinal studies and further clinical-pathological correlations. In AD patients, a reorganization of cortical functions has been reported since the early stages, likely due to the occurrence of a frontal and medial shift of the “center of gravity” of the TMS-based cortical motor map representations [112, 113]. A similar pattern has been shown also in subcortical ischemic VaD, which identifies a homogenous subtype of patients characterized by insidious onset, gradual course, and relatively slow progression, which make them hard to differentiate them from AD patients [114].

Although much less is known, plastic phenomena have been also reported to take place also in VaD. While exploring the relationship between excitability and plasticity in subcortical ischemic VaD, a cross-sectional study found that M1 had enhanced excitability in both AD and subcortical ischemic VaD patients, and more interestingly, M1 was plastically rearranged in both groups [92]. The results demonstrated indeed a functional cortical reorganization of all patients, with a slightly smaller frontal shift in the center of gravity for subcortical ischemic VaD compared to AD. A direct correlation between parameters of cortical excitability and those associated with the topographic shift of cortical maps was also noted [92]. Authors hypothesized that partially overlapping electrophysiological mechanisms probably act in the same manner in both VaD and AD, although they may differ both in location (subcortical vs. cortical) and origin (vascular vs. degenerative). Therefore, these disorders might share a common neurophysiological platform represented by a progressive neuronal loss in the motor areas in AD and a vascular disconnection in the white matter in subcortical ischemic VaD [115]. Eventually, these alterations will promote a functional brain rearrangement allowing to preserve motor programming and execution [84, 85].

Neurochemically, the reduction of rMT in both VaD and AD might represent a marker of impaired glutamatergic transmission, with an imbalance between non-NMDA and NMDA activity [116, 117]. Coherently, enhanced cortical excitability has been observed after the administration of an NMDA antagonist [118]. However, the facilitation of cortico-spinal outputs might also be caused by reduced intracortical inhibition [33]. Indeed, an increased GABA release may be a response to an overactivation of glutamate as part of the neuronal defense mechanisms leading to the compensation for excitotoxicity [119]. However, the studies here reviewed did not find significant changes of the TMS-related measures of inhibition, such as cSP, iSP, and SICI, while a significant SAI reduction was found in subcortical ischemic VaD [89]. In a different study, however, the reduction of SAI was noted in AD but not in VaD, apart from 25% of VaD patients that probably had a mixed dementia [88]. Even microbleeds in subcortical ischemic VaD might to have an impact on SAI-related cholinergic pathways, which was independent of the WMLs extent and ischemic stroke [91].

### 3.3. Vascular Depression

TMS studies are in line with the other findings in classifying vascular depression as a distinct nosologic entity, different from early-onset MDD [94]. In vascular depression, depressive symptoms, rather than signs of a primary disease status, are part of the wide spectrum of clinical presentations of the subcortical cerebrovascular disease [93]. Another difference between geriatric vascular depression and early-onset MDD is the enhancement of ICF observed only in former [93, 94]. According to the vascular depression hypothesis, this finding may imply that the disruption of the frontal-striatal circuits caused by vascular lesions may predispose, precipitate, or perpetuate a late-life depression [120].

However, from a neurophysiological perspective, very little is known on plasticity preserving cognitive functions in geriatric depression. By investigating the evolution of neurophysiological parameters in nondepressed patients with mild VCI and those with vascular depression, it has been shown that only nondepressed patients had a high level of ICF at the initial TMS evaluation [95]. At follow-up, a glutamate-related enhanced plasticity may have taken place in nondepressed patients that might be protective against cognitive deterioration, giving also cues on the possible role played by the late-life depression in the progression of VCI. Further, reduced rMT in both patient groups at follow-up points to the glutamatergic neurotransmission involvement. However, no specific change of neurophysiological parameter correlated with cognitive decline in depressed patients, suggesting that cognitive deterioration in vascular depression might be related to the subcortical lesion load or to the lack of compensatory cortical inputs [95].

### 3.4. CADASIL

The mutations in the *Notch3* gene on chromosome 19 causes CADASIL, that manifests with progressive cognitive decline till dementia, migraine, psychiatric disorders, and cerebral ischemic events. For this reason, it represents a genetic model of VaD that is interesting to study from a neuropsychologic and electrophysiological point of view [121].

In the first TMS study, a reduction in rMT and SAI [96] was found and attributed to the disruption of different cortical-subcortical circuits caused by vascular lesions and locations [122], such those affecting the external capsule [123]. Regarding SAI, the significant reduction observed in both AD and CADASIL may be due to the involvement of different pathways, in that the L-3,4-dihydroxyphenylalanine (L-DOPA) administration was able to restore SAI only in AD [99], thus also providing therapeutic insights.

CADASIL patients also present an impaired sensory-motor plasticity induced by PAS [97]. Further, an association between WMLs load and lowered fractional anisotropy, along with an abnormal enhancement of LTP-like plasticity induced by PAS, has been observed particularly in the frontal commissural fibers. The authors' suggestion was that the increase in cortical plasticity might compensate the deterioration of cognitive and motor functions [97]. However, older patients with impaired cognition manifested opposite results, with a lower PAS-induced cortical plasticity, as well as a reduction of SAI and ICF [98]. In this study, a lower LTP-like plasticity in a stage of overt cognitive disorder may have failed in the compensatory mechanisms [98].

### 3.5. Repetitive TMS in Vascular Cognitive Impairment

Several rTMS studies, although methodologically heterogeneous, have shown that specific paradigms of stimulation might improve cognitive performance and have been proposed as a possible alternative to conventional neuroleptic therapy to behavioral symptoms of dementia. This is of particular interest because current pharmacological treatments suffer of significant limitations, such as nonspecific effects, insufficient tailoring to the individual, and moderate-to-severe adverse effects [124]. In this context, the target for an ideal rTMS treatment would be: (i) modulation of activity specifically in the targeted cortex, (ii) modulation of activity in a dysfunctional network, (iii) restoration of adaptive balance in a disrupted network, (iv) guiding plasticity for best outcome, and (v) suppression of maladaptive changes for functional advantage.

In a randomized controlled pilot study on patients with subcortical ischemic vascular disease and a clinical diagnosis of mild VCI, high-frequency rTMS over the left dorsolateral prefrontal cortex (DLPFC) induced a long-lasting improvement of the executive performance, likely due to an indirect activation of the midbrain monoaminergic neurons (dopamine) and/or of the brainstem (noradrenaline and serotonine) and their cortical and subcortical targets [100]. In the same patients, rTMS was able to alleviate depressive symptoms, suggesting a potential application even in individuals with vascular depression, although WMLs and global vascular risk factors were predictors of poor response [125].

Few years later, a randomized, controlled, crossover study on 7 mild VCI patients [101] stimulated the left DLPFC and the left M1 both at low- and high-frequency rTMS for 4 sessions (two at 1 Hz and two at 10 Hz). The authors found a significant improvement in the Stroop color-word interference test after the stimulation of the DLPFC but not the M1. An improvement was also noted in the digit symbol subtest of the revised Wechsler Adult Intelligence Scale after rTMS, regardless of the stimulation site [101].

### 3.6. Repetitive TMS in Vascular Depression

Based on the FDA approval for the treatment of drug-resistant MDD in adults [126] and according to the view that depressed patients exhibit a significant interhemispheric asymmetry in motor cortex excitability (i.e., lower excitability of the left hemisphere) [34], two main rTMS protocols, i.e., high-frequency rTMS (5-20 Hz) over the left DLPFC and low-frequency rTMS (1 Hz) on the right DLPFC [127], have been evaluated. The protocol using the high-frequency rTMS [128] reached a remission rate up of 15% in the “real” (treated) stimulation group with respect to 5% of the “sham” (simulated) stimulation group [129].

Globally, rTMS seems to be less effective in late-onset patients with geriatric depression [130, 131], probably due to the brain atrophy (especially in the frontal lobes) and ischemic WMLs (especially in the prefrontal areas) characterizing this age group, both disrupting the connections between DLPFC and subcortical areas underlying mood and affect control [132]. Nevertheless, an earlier analysis [133] did not find age as a significant predictor of response, whereas positive predictors were a shorter duration of the current depressive episode (<2 years) and the degree of treatment resistance (≤1 treatment failure vs. >1).

In the attempt of exploiting rTMS as a therapeutic option for vascular depression, a small open trial showed that 10 sessions of high-frequency rTMS applied over the left DLPFC improved not only verbal fluency, visuospatial memory, and delayed recall but also depressive symptoms [102]. In a larger prospective randomized sham-controlled study, high-frequency rTMS over the same cortical region successfully treated depressive symptoms and increased both response and remission rates [103]. These results suggested that rTMS may modulate both cognitive ability and depressive symptoms, probably by activating different but closely spaced neural networks. Preliminary findings were confirmed by two subsequent randomized trials, one that combined rTMS with citalopram treatment [104] and one by using electroencephalography (EEG) in the follow-up period [105]. The studies showed significant differences in response and remission rates of depressive symptoms between active and sham groups, favoring the “real” stimulation. The second study also found that the increased “low-theta” band power in the subgenual cingulate cortex predicted the response to rTMS [105].

Finally, low-frequency rTMS over the right DLPFC was tested in a patient with drug-resistant depression and cerebral amyloid angiopathy, which is a chronic neurovascular disorder characterized by a progressive amyloid-*β* fibril deposition within the wall of cerebral blood vessels, eventually leading to hemorrhagic events and dementia. Stimulation intensity was set to 110% of the rMT, and rTMS was applied at 1 Hz for 1,600 pulses per day for 3 weeks. A long-lasting decrease in depression rating scales was noted, thus opening the way for the treatment of depression in other forms of cerebrovascular and degenerative diseases [134].

### 3.7. Translational Considerations

To date, the prediction of dementia onset and progression is beyond the possibilities of conventional tools. However, differently from AD and other degenerative disorders, VaD can be slowed, delayed, or even avoided through a careful prevention and control of vascular risk factors [135]. Besides the prevention of vascular accidents, maintaining the functional status in the elderly is a further key factor in the prevention and management of VCI.

Because of the VCI's heterogeneous construct, the selection of appropriate outcome measures to employ in pharmacological trials is of particular importance. In this context, the early discovery of new therapeutic targets would lead to a better prevention and treatment of VaD, and accordingly with the reviewed literature, TMS can be of help [33]. An enhancement of cortical plasticity might be induced to counteract cognitive decline, and the evaluation of where and how much this happens in different patients' subpopulations may shed light on the pathophysiological bases of decline or preservation of cognition [115].

Although a single TMS measure cannot be used to diagnose VCI, collectively the parameters of interest may act as footprints of VCI pathophysiology. Moreover, TMS can help to identify different profiles of cortical excitability for VCI subtypes and for the prediction of the “brain at risk” to convert into an overt VaD [28, 31, 95]. These findings will also support the study design of trials to test new drugs and novel nonpharmacological approaches. Finally, clinicians can exploit TMS in patients with overt dementia for the selection of the response to specific drugs [110], and the efficacy of treatment can be maximized by selecting the patients on the basis of putative neurophysiological markers.

Neurotrophins have an important role in the response to vascular damage and in stroke recovery [136, 137], and their release and modulation may also be behind the mechanisms of action of noninvasive brain stimulation in dementia. Several murine models of VaD have been used for testing rTMS [138], showing that low-frequency rTMS positively impact on cognitive deficit by upregulating the release of the hippocampal brain-derived neurotrophic factor (BDNF) and the expression of the NMDA glutamate receptor [139]. A different study found that increased expression in the *Bcl-2* gene and a decrease in the *Bax* gene led low-frequency rTMS to be effective in learning and memory, as well as in the protection of pyramidal cells from apoptosis and in the promotion of hippocampal synaptic plasticity [140]. Moreover, rTMS significantly improved learning and memory and increased acetylcholinesterase and choline acetyltransferase activity, the density of cholinergic neurons, and the number of BDNF-immunoreactive cells at the level of hippocampal CA1 region [141]. Finally, in VaD rats, synaptic plasticity showed to be synergic with mesenchymal stem cells transplantation and with the promotion of autophagy [142]. However, the effectiveness of rTMS as VCI disease-modifying therapy in humans deserves further translational considerations, larger samples size, and well-controlled investigations [143].

Similarly, the clinical efficacy of rTMS on the cognitive aspects of vascular depression is still a matter of debate. It cannot be excluded, indeed, that cognitive improvement might be the consequence of an indirect effect on depressive symptoms rather than an improvement of cognition *per se*. In this context, while findings on rTMS in vascular depression are still limited and a conclusive evidence is yet to be reached, rTMS data in MDD (which is often associated with cognitive changes, especially executive dysfunction) are much more robust [144]. In MDD, the treatment-induced response did not seem to be directly related to a relief from depression or other treatment variables, thus suggesting that improvement of cognition and mood may follow different mechanisms [145]. Based on earlier controlled studies [146–148], improvement in both verbal fluency and visuospatial memory suggests that rTMS may enhance specific aspects of cognition independently from positive mood changes through a general alerting effect or a learning facilitation [102]. Moreover, since previous investigations did not find significant correlations between cognitive functioning and depression scores [149–151], it has been proposed that rTMS might independently modulate cognitive abilities and depression symptoms by activating different neural pathways and brain regions. In addition, in a pilot study on treatment-resistant depressed patients [151], left frontal high-frequency rTMS was associated with better performance of tests evaluating frontal lobe abilities and reduction in depression severity. The authors hypothesized that the cognitive improvement could be due to a direct or indirect (i.e., transsynaptic) modulation of the DLPFC [151], probably secondary to the activation of monoaminergic neurons in the midbrain (dopamine) or in the brainstem (noradrenaline and serotonin) and their cortical and subcortical targets [152, 153].

Lastly, it was demonstrated that rTMS not only improved executive dysfunction in MDD patients but also restored the interhemispheric asymmetry of rMT and ICF, thus implying that specific electrocortical changes may correlate to executive functions, both before and after treatment [154]. Although the pattern of motor cortex excitability in vascular depression differs from that previously reported in MDD and is similar to that of patients with subcortical vascular disease [28], the clinical presentations of these patients are similar, i.e., psychomotor retardation, difficulties at work, apathy, lack of insight, and executive dysfunction. This may suggest that, in vascular depressed patients, the enhancement of ICF could play a compensatory glutamate-mediated role in response to vascular damage of the frontal cortical-subcortical circuits implicated in mood-affect regulation and cognition [94]. Nevertheless, as mentioned above, the effects of rTMS on cognitive functioning can depend on additional factors (e.g., the paradigms used and the parameters studied), and it is not always observed [155], thus warranting further evidence in both MDD and vascular depression.

At this stage, it is not certain that the findings of the studies we reviewed have a direct impact in the daily decision-making algorithm of VCI patients' care, although they suggest that TMS can help to screen populations at risk. Once the population at risk is identified, a careful prevention and vascular risk factors control can be achieved more easily. Further longitudinal studies combining TMS with other neurophysiological techniques, such as high-density EEG and multimodal evoked potentials, as well as with advanced structural and functional neuroimaging (such as diffusion tensor imaging and functional MRI) and serum or CSF analysis will clarify the impact of cognitive and mood deficits on VCI plasticity.

**Figure 3:**
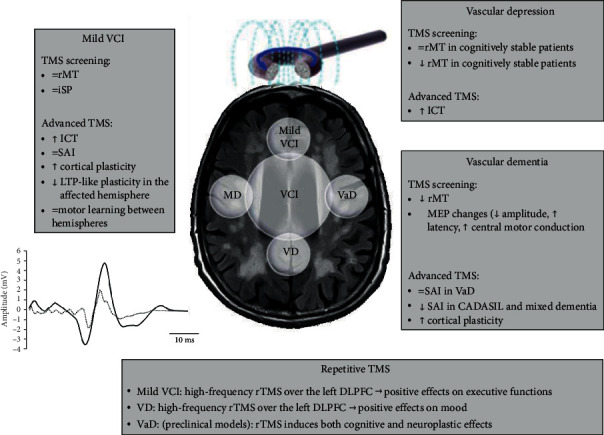
TMS findings, proposed diagnostic algorithm, and main rTMS effects in VCI. Legend (in alphabetic order): CADASIL: cerebral autosomal dominant arteriopathy with subcortical infarcts and leukoencephalopathy; DLPFC: dorsolateral prefrontal cortex; ICF: intracortical facilitation; iSP: ipsilateral silent period; LTP: long-term potentiation; MD: mixed dementia; MEP: motor evoked potential; rMT: resting motor threshold; rTMS: repetitive transcranial magnetic stimulation; SAI: short-latency afferent inhibition; TMS: transcranial magnetic stimulation; VaD: vascular dementia; VCI: vascular cognitive impairment; VD: vascular depression.

Figure 3 illustrates the TMS findings in VCI, proposes a diagnostic algorithm, and summarizes the main rTMS effects.

### 3.8. Critical Aspects, Possible Solutions, and Future Research

A major limitation in the implementation of the studies employing noninvasive brain stimulation in VCI is the relatively small sample sizes that make the generalization of these results to large populations troublesome. The same holds for the difficulty to recruit enough elderly healthy controls without neuroimaging evidence of cerebrovascular disease.

Second, the relatively low spatial resolution of TMS often determines the lack of systematic correlation between the pattern of cortical excitability and the anatomical distribution and severity of vascular lesions. Combining TMS with advanced imaging, neuronavigational systems, and other electrophysiological techniques may overcome this issue.

Third, although the TMS-related measures of cortical excitability are sensitive to the “global weight” of many neurotransmitters, so far we do not have more detailed information linking TMS findings with specific cognitive or behavioral changes [54, 111]. In this context, hypothesizing the presence a specific “signature” characteristic of VCI patients could be risky given the paucity of previous data and the difficulty that similar approaches are encountered in other dementing conditions, such as the non-AD dementias [33]. Additionally, even in the absence of evident motor deficit, vascular lesions significantly contribute to degenerative dementias and their progression. Therefore, it cannot be excluded that some of the enrolled patients had a mixed dementia rather than a pure VaD. In other words, TMS profile alone is not currently capable of distinguishing VaD from AD [115].

It should also be noted that antithrombotic agents, oral antidiabetic therapy, antihypertensive drugs, and statins, commonly prescribed to elders, might affect the measures of cortical excitability and their response to rTMS treatments [156, 157]. Thus, both TMS and rTMS studies need to consider this possible confounding factor.

Finally, for an adequate definition of sensitivity and specificity, the individual TMS measures in all patients and controls would be necessary. Besides, the estimation of the number of false positives would require an independent follow-up allowing the assessment of the cognitive status. Those requirements have been met only by a few studies, so that the next applications of TMS in VCI need methodological improvements and higher standardization levels.

Regarding rTMS, it is relatively expensive and requires technical expertise. Moreover, the magnetic coil must to be held still, and sham stimulation and operator blindness are often difficult. The majority of reported investigations are open-label or uncontrolled, and the treatment response could be affected by changes in brain morphology (e.g., cortical atrophy or CSF distribution). Moreover, determining the most appropriate target for stimulation is often challenging, and inferring to what extent cortical response characteristics of the motor system are representative of other brain areas is often speculative. Finally, there is a wide range of TMS parameters and rTMS settings that need to be considered in these applications.

Possible solutions may consist of [158]: (i) fully report of the results all rTMS trials, including negative findings; (ii) more studies in healthy individuals or in those with mild disease, thus allowing finessing of stimulation parameters and establishing the tolerability of protocols; (iii) further studies on the etiological models of dementia, including preclinical ones, thus aiding the choice of stimulation site and other technical set up; (iv) optimization of the treatment efficacy through methods of stratification, where patients are selected on the basis, for instance, of neuropsychological, electrophysiological, or genetic markers; and (v) use of novel methodological factors that can increase the stimulation efficacy, as well as the combination of rTMS with objective outcome measures (e.g., those derived from EEG, CSF, or MRI).

## 4. Conclusions

Overall, there is a mounting interest towards new diagnostic and therapeutic tools for cognitive assessment and rehabilitation in dementia, including VCI. Current data, although obtained from heterogeneous studies, have revealed that TMS and rTMS can provide, respectively, valuable diagnostic clues and induce beneficial effects on some cognitive domains and neuropsychiatric manifestations. Challenges still exist in terms of appropriate patient selection and optimization of the stimulation protocols. Recent findings from animal models are exciting, but their clinical significance needs to be validated. Together with the clinical exam, psychocognitive assessment, and neuroimaging, a systematic TMS evaluation of VCI patients can aid the diagnostic process, enhance the therapeutic arsenal, and predict the prognosis.

## Figures and Tables

**Table 1:** TMS studies in patients with vascular cognitive impairment.

VCI subtype	Study, year	Study design	Patients *n*	Main findings
Mild VCI	Bella et al., 2011 [30]	Cross-sectional	10	↑ Intracortical excitatory neuronal circuits
	Bella et al., 2013 [31]	Case-control	9	↑ Excitability during the progression of VCI
	Lanza et al., 2013 [83]	Cross-sectional	15	= Transcallosal inhibitory functioning, unlike AD and mild cognitive impairment
	List et al., 2013 [84]	Cross-sectional	20	↑ Cortical plasticity as a compensatory mechanism
	List et al., 2014 [85]	Cross-sectional	12	↓ LTP-like plasticity in the affected hemisphere = Motor learning between hemispheres, maybe due to a GABA-B effect in the affected side
	Bella et al., 2016 [86]	Cross-sectional	25	Central cholinergic pathway not clearly affected

Vascular dementia	Alagona et al., 2004 [87]	Cross-sectional	20 AD 20 SIVD 20 HC	↓ Motor threshold in SIVD compared to AD and HC
	Di Lazzaro et al., 2008 [88]	Cross-sectional	12 VaD 12 AD 12 HC	= Short-latency afferent inhibition in VaD patients and significantly reduced in AD
	Nardone et al., 2008 [89]	Cross-sectional	20 SIVD 25 HC	↓ Mean short-latency afferent inhibition in patients
	Pennisi et al., 2011 [90]	Cross-sectional	20 VaD 20 mild VCI	↑ Cortical excitability in demented patients only
	Nardone et al., 2011 [91]	Cross-sectional	28	Microbleeds on cholinergic function are independent of white matter lesion extent and ischemic stroke
	Guerra et al., 2015 [92]	Cross-sectional	7 VCI 9 AD	↑ Excitability and plasticity in AD and VaD Hyperexcitability promoted plasticity

Vascular depression	Bella et al., 2011 [93]	Cross-sectional	15 MDD 10 non-depressed	Neurophysiology of vascular depression differs from MDD, and it is similar to that of subcortical ischemic vascular disease
	Concerto et al., 2013 [94]	Cross-sectional	11 depressed 11 MDD	Different patterns of cortical excitability between late-onset vascular depression and early-onset nonvascular MDD
	Pennisi et al., 2016 [95]	Case-control	16 MDD 11 nondepressed	↑ Risk of dementia in vascular depression, probably due to subcortical vascular burden or to the lack of compensatory functional cortical changes

CADASIL	Manganelli et al., 2008 [96]	Cross-sectional	10 CADASIL 10 HC	↓ Short-latency afferent inhibition in patients ↓ Resting motor threshold significantly reduced in patients
	List et al., 2011 [97]	Cross-sectional	12 CADASIL 10 HC	↑ Cortical plasticity in patients compared to HC
	Palomar et al., 2013 [98]	Cross-sectional	10	Acetylcholine and glutamate were involved Abnormal sensory-motor plasticity correlated with cognition
	Nardone et al., 2014 [99]	Cross-sectional	8 CADASIL 8 AD	↓ Cholinergic functioning, with restoration by L-3,4-dihydroxyphenylalanine in AD group only

Legend (in alphabetical order): AD: Alzheimer's disease; CADASIL: cerebral autosomal dominant arteriopathy with subcortical infarcts and leukoencephalopathy; GABA: gamma-aminobutyric acid; HC: healthy controls; LTP: long-term potentiation; MDD: major depressive disorder; *n*: patients' number; SIVD: subcortical ischemic vascular disease; VaD: vascular dementia; VCI: vascular cognitive impairment; ↑: increase/enhancement; ↓: decrease/reduction; =: no significant change/modification.

**Table 2:** Repetitive TMS studies in patients with vascular cognitive impairment.

VCI subtype	Study, year	Study characteristics	Main findings
Mild VCI	Rektorova et al., 2005 [100]	Type of study: randomized, controlled, blinded, crossover Subjects: 7 Type of coil: figure-of-eight coil (7 cm diameter) Stimulation site: left DLPFC (active), left M1 (control) Stimulation frequency: 10 Hz Intensity: 100% rMT Length: 3 rTMS blocks, separated by a 10 min interval; in each block, fifteen 10-pulse trains, each of 1 s duration, delivered with an intertrain interval of 10 s Duration: 1 session Total number of pulses delivered: 450	Significant positive effect of active stimulation on the Stroop color-word interference test
	Sedlackova et al., 2008 [101]	Type of study: randomized, controlled, blinded, crossover Subjects: 7 Type of coil: figure-of-eight coil (7 cm diameter) Stimulation site: left DLPFC (active), left M1 (control) Stimulation frequency: 1 Hz; 10 Hz Intensity: 100% rMT Length: for the 10 Hz stimulation: 3 rTMS blocks, separated by a 10-minute interval; in each block, fifteen 10-pulse trains, each of 1 s duration, delivered with an intertrain interval of 10 s; for the 1 Hz stimulation: continuous Duration: 4 sessions (two at 1 Hz and two at 10 Hz) Total number of pulses delivered: 450 at 10 Hz; 1,800 at 1 Hz	Significant improvement in the Stroop color-word interference test after the stimulation of DLPFC but not M1; improvement in the digit symbol subtest of the Wechsler Adult Intelligence Scale-revised after rTMS, regardless of the stimulation site. No measurable effect in any other neuropsychological test

Vascular depression	Fabre et al., 2004 [102]	Type of study: open trial Subjects: 11 Type of coil: figure-of-eight coil Stimulation site: left prefrontal cortex Stimulation frequency: 10 Hz Intensity: 100% rMT Length: twenty 8 s trains, with 52 s intertrain intervals Duration: 10 sessions over two weeks	Five out of 11 patients responded to rTMS in terms of clinically meaningful improvement in HDRS scores, with a decrease by at least 25% from baseline; improvement of verbal fluency, visuospatial memory, and delayed recall
	Jorge et al., 2008 [103]	Type of study: prospective, randomized, sham-controlled Subjects: 92, randomized in active (48) and sham (44) groups; experiment 1: two groups of 15 patients each; experiment 2: 33 “real” patients and 29 sham patients Type of coil: figure-of-eight coil (7 cm diameter) Stimulation site: left DLPFC Stimulation frequency: 10 Hz Intensity: 110% rMT Length: 30 minutes Duration: 10 sessions (experiment 1), 15 sessions (experiment 2), 6 s period of stimulation, with a total of 20 trains separated by 1 min pauses, over 10 days Total number of pulses delivered: 12,000 (experiment 1); 18,000 (experiment 2)	Experiment 1: significant decrease in HDRS scores for real stimulation compared to sham; experiment 2: significant decrease in HDRS scores, increase in response rates, and remission rates for real stimulation compared to sham. Response rates to rTMS negatively correlated with age and positively correlated with higher frontal gray matter volume
	Robinson et al., 2009 [104]	Same patients and protocol of the experiment 2 of the study by Jorge and colleagues (2008) [103]. After rTMS or sham treatment, all responders were given citalopram for 9 weeks	Among the 33 “real” patients, 13 responded (>50% decrease in HDRS score); among them, 9 patients continued to be responders whereas the reaming 4 had a relapse of depression during the course of citalopram treatment
	Narushima et al., 2010 [105]	Type of study: prospective, randomized, sham-controlled Subjects: 43, randomized in “real” (32 patients) and “sham” (11 patients) groups Type of coil: figure-of-eight coil (7 cm diameter) Stimulation site: left DLPFC Stimulation frequency: 10 Hz Intensity: 110% rMT Duration: 10 sessions, 6 s period of stimulation, with a total of 20 trains separated by 1 min pauses, performed over 10 days Total number of pulses delivered: 12,000 (12 patients)–18,000 (31 patients)	Significant difference in the response and remission rate of the HDRS scores between active and sham groups, in favor of the “real” stimulation group; increased low-theta power in the subgenual cingulate predicted the response to rTMS

Legend (in alphabetical order): DLPFC: dorsolateral prefrontal cortex; HDRS: Hamilton depression rating scale; M1: primary motor cortex; rMT: resting motor threshold; rTMS: repetitive transcranial magnetic stimulation; VCI: vascular cognitive impairment.

## Data Availability

No data were used to support this study.
